# AI-driven proteomics: unlocking early cardiac detection in POEMS syndrome

**DOI:** 10.1097/MS9.0000000000003888

**Published:** 2025-09-15

**Authors:** Hamna Fatima, Eman Fatima, Maliha Khalid, Muhammad Talha, Aminath Waafira

**Affiliations:** a Department of Medicine, Nishtar Medical College and Hospital: Multan, Punjab, Pakistan; b Department of Medicine, Allama Iqbal Medical College, Lahore, Pakistan; c Department of Medicine, Jinnah Sindh Medical University, Karachi, Pakistan; d Department of Medicine, King Edward Medical University, Lahore, Pakistan; e School of Medicine, The Maldives National University, Malé, Maldives

**Keywords:** artificial intelligence, cardiac biomarkers, POEMS syndrome, proteomics

## Abstract

POEMS syndrome is a rare paraneoplastic multisystem disorder with significant morbidity when cardiac involvement occurs, yet diagnosis is often delayed until advanced stages. Recent advances in artificial intelligence (AI)-driven proteomics offer a transformative approach for early detection. Cardiac-specific biomarkers such as N-terminal pro-B-type natriuretic peptide, vascular endothelial growth factor, and growth differentiation factor-15 correlate with myocardial stress and fibrosis, providing diagnostic clues even when imaging is inconclusive. AI-enhanced mass spectrometry can identify previously undetectable protein isoforms and post-translational modifications linked to cardiac pathology, while machine learning algorithms facilitate rapid, high-accuracy interpretation of complex datasets. Despite limitations such as the rarity of cases, heterogeneous presentations, and integration challenges, standardized protocols, collaborative data sharing, and biomarker validation could establish AI-driven proteomics as a pivotal tool for improving early cardiac detection and prognosis in POEMS syndrome.

*To the Editor,*

POEMS syndrome is a rare paraneoplastic multisystem disease characterized by polyneuropathy, organomegaly, endocrinopathy, monoclonal gammopathy, and skin changes. Although rare, it still has an estimated prevalence of <0.3 cases per 100 000^[1]^. It leads to irreversible organ damage, especially when cardiac involvement is present. Cardiac complications, such as cardiomyopathy, pericarditis, pulmonary hypertension, and pericardial effusion, often present late and are major contributors to morbidity. Many artificial intelligence (AI)-powered proteomics tools have been developed, enabling error-free and high-quality protein profiling and identifying cardiac pathology with unprecedented sensitivity^[2]^.

Recent developments have shown novel cardiac-specific protein biomarkers in POEMS patients, such as N-terminal pro-B-type natriuretic peptide (NT-proBNP), vascular endothelial growth factor (VEGF), and growth differentiation factor-15. These factors correlate with cardiac stress and serve as an early detection tool, particularly in subclinical stages where imaging may be inconclusive. AI-enhanced mass spectrometry has further amplified this landscape by identifying previously undetectable isoforms and post-translational modifications linked to myocardial fibrosis. The use of machine learning (ML) algorithms allows rapid interpretations of complex proteomic datasets. Thus, AI-derived proteomics holds a revolutionary potential for POEMS syndrome diagnostics^[3]^.

Current literature has highlighted the importance of protein biomarkers in diagnosing cardiac involvement in this disorder. One of the most important biomarkers, VEGF, increases vascular permeability of the coronary microvasculature. It causes fluid accumulation, hence leading to left ventricular hypertrophy and cardiac failure. In a recent study, serum VEGF was markedly elevated at 800 pg/ml (normal range 0–142 pg/ml) and its levels decreased significantly following the treatment and resolution of symptoms^[4]^. NT-proBNP, along with other biomarkers, also helps in the prognosis of cardiac disease. In congestive heart failure associated with POEMS syndrome, NT-proBNP levels as high as 12 631 pg/ml have been reported^[5]^. Moreover, AI-driven tools enhance the utilization and efficacy of these protein biomarkers.

Since there are ongoing developments, there are some limitations involved. The most important among those is that this syndrome is a rare disorder, due to which there is not much data available. The heterogeneity in the clinical presentation of patients, integration issues, ethical concerns, cost limitations, and unverified biomarkers also limit the process. In addition to that, ML and AI-based tools require validation before they can be practically applied in the diagnosis and prognosis of this clinical entity^[6]^. Our work is in line with the TITAN Guidelines on the need for transparency in AI use in healthcare^[7]^.

In conclusion, AI-driven proteomics and the discovery of novel cardiac biomarkers will be revolutionary in detecting the cardiac association of this rare multisystem disorder. There should be standardized protocols, collaborative data sharing, and validation of protein biomarkers for improving diagnostic accuracy. In this way, it will be possible to fully realize the diagnostic and prognostic potential of AI-driven proteomics in the context of POEMS syndrome.

## Data Availability

No data were generated for this manuscript.

## Research registration unique identifying number (UIN)

Name of the registry: Not Applicable. Unique Identifying number or registration ID: Not Applicable. Hyperlink to your specific registration (must be publicly accessible and will be checked): Not Applicable.

## Peer and provenance statement

This article was not commissioned and was externally peer-reviewed.
